# Prevalence of *bla*_NDM_, *bla*_PER_, *bla*_VEB_, *bla*_IMP_, and *bla*_VIM_ Genes among *Acinetobacter baumannii* Isolated from Two Hospitals of Tehran, Iran

**DOI:** 10.1155/2014/245162

**Published:** 2014-07-15

**Authors:** Fatemeh Fallah, Maryam Noori, Ali Hashemi, Hossein Goudarzi, Abdollah Karimi, Soroor Erfanimanesh, Shadi Alimehr

**Affiliations:** ^1^Pediatric Infections Research Center, Mofid Children Hospital, Shahid Beheshti University of Medical Sciences, Tehran, Iran; ^2^Department of Microbiology, Shahid Beheshti University of Medical Sciences, Tehran, Iran

## Abstract

*Background and Objectives*. The aim of this study was to determine the frequency of *bla*_NDM_, *bla*_PER_, *bla*_VEB_, *bla*_IMP_, and *bla*_VIM_ type genes among *A. baumannii* isolates from hospitalized patients in two hospitals in Tehran, Iran. *Patients and Methods*. Antibiotic susceptibility tests were performed by Kirby-Bauer disc diffusion and Broth microdilution methods. The frequency of MBL (metallo-beta-lactamase) and ESBL (extended-spectrum-beta-lactamase) producers was evaluated by CDDT. The *β* *-lactamases* genes were detected by PCR and sequencing methods. *Results*. The resistance of *A. baumannii* isolates against tested antibiotics was as follows: 103 (95.4%) to ceftazidime, 108 (100%) to cefotaxime, 105 (95.7%) to cefepime, 99 (91.7%) to imipenem, 99 (91.7%) to meropenem, 87 (80.6%) to amikacin, 105 (97.2%) to piperacillin, 100 (92.6%) to ciprofloxacin, 103 (95.4%) to piperacillin/tazobactam, 44 (40.7%) to gentamicin, 106 (98.1%) to ampicillin/sulbactam, 106 (98.1%) to co-trimoxazole, 87 (80.6%) to tetracycline, and 1 (1.8%) to colistin. Using combined disk diffusion test, 91 (84.2%) and 86 (86.86%) were ESBL and MBL producers, respectively. The prevalence of *bla*_PER-1_, *bla*_VEB-1_, *bla*_IMP-1_, and *bla*_VIM-1_ genes was 71 (78.03%), 36 (39.5%), 3 (3.48%), and 15 (17.44%), respectively. *Conclusions*. The prevalence of ESBLs and MBLs-producing *A. baumannii* strains detected in this study is a major concern and highlights the need of infection control measures.

## 1. Background

Multidrug-resistant bacterial strains have emerged as the causes of nosocomial infections worldwide. Recently, pandrug-resistant (PDR) bacterial strains, which are resistant to all antibacterial agents except the polymyxins and tigecycline, and extensively drug-resistant (XDR) bacterial strains, which are resistant to all antibacterial agents, were isolated from hospital-acquired infections. There is a huge risk of these “superbugs” extending into the community and threatening public health [1].* A. baumannii* is an important cause of nosocomial infections and a leading cause of mortality and morbidity among hospitalized patients and has been associated with a wide variety of diseases in hospitalized patients in the intensive care units (ICU) [2]. Nowadays, increasing drug resistant rate among* A. baumannii* strains is a major concern worldwide [3]. The most common mechanism of resistance is the production of *β*-lactamases, including enzymes of Ambler classes A, D, and B, with their genes being often associated with mobile genetic elements such as plasmids [4]. Carbapenem resistance caused by acquiring the MBLs is considered to be more serious than other resistance mechanisms because MBLs can almost hydrolyse all beta-lactam antibiotics except monobactams [3]. Furthermore, the MBL-encoding genes located on integrons can be disseminated easily from one bacterium to another. Many MBLs have been found in* A. baumannii*, including imipenemase (IMP), São Paulo metallo (SPM), Verona integron-encoded metallo-beta-lactamases (VIM), Seoul imipenemase (SIM), Japan, Kyorin University Hospital imipenemase (KHM), German imipenemase (GIM), New-Delhi metallo-beta-lactamase (NDM-1), and Australian imipenemase (AIM) [5, 6]. The IMP-type enzymes, first detected in Japan in the late 1980s, have since been reported worldwide in* Enterobacteriaceae* and in Gram-negative nonfermenters (mostly in* P. aeruginosa* and* Acinetobacter spp.*). More than 20 different IMP allotypes have been described, belonging to various sublineages [4]. The different IMP types often have a defined area in the globe, however, some of which (e.g., IMP-1, IMP-4, and IMP-7) were discovered in different areas which shows their potential for intercontinental dissemination. The VIM-type *β*-lactamase (Verona integron-encoded metallo-*β*-lactamases) was first described in a multidrug-resistant* P. aeruginosa* strain in Italy during the 1990s and has since been reported worldwide. More than 33 different VIM allotypes are described [4].

New-Delhi-metallo-beta-lactamase (NDM-1), a new type of MBL, was first detected in two* K. pneumoniae* and* Escherichia coli* strains isolated from a Swedish patient who was admitted to a hospital in New Delhi, India. In recent years, the emergence and dissemination of NDM-1-producing isolates have been reported in several countries, including USA, Canada, Sweden, UK, Austria, Belgium, France, The Netherlands, Germany, Japan, Africa, Oman, and Australia [4]. NDM-producing bacteria are commonly resistant to almost all groups of antibiotics, including fluoroquinolones, aminoglycosides, and *β*-lactams (especially carbapenems) but are susceptible to colistin and sometimes tigecycline. The *bla*_NDM-1_ gene has been detected on different large plasmids, which were readily transferable among bacteria, making NDM-1-producing bacteria a serious clinical and public health threat [7]. Extended spectrum beta-lactamases (ESBLs) are a rapidly evolving group of *β*-lactamase enzymes produced by some bacteria. These enzymes have the ability to hydrolyze cephalosporins and aztreonam but are inhibited by *β*-lactamase inhibitors such as clavulanic acid [8]. ESBL genes are often located on plasmids and many of them are derived from mutations in TEM (Temoneira) genes and SHV (Sulphydryl variable) detected by amino acid substitutions around the active site. Apart from TEM and SHV ESBL types, isolates may additionally produce CTX-M (Cefotaximase-Munchen) *β*-lactamase [8]. Other clinical types include *bla*_KPC_,  *bla*_VEB_,  *bla*_PER_,  *bla*_BEL-1_,  *bla*_BES-1_,  *bla*_SFO-1_,  *bla*_TLA_, and *bla*_BIC_ [9]. But in recent years, new families of extended-spectrum-beta-lactamases have also emerged all over the world, including PER (for Pseudomonas extended resistance) and VEB (for Vietnamese extended-spectrum-beta-lactamase) families [10].

## 2. Objectives

The aim of this study was to determine the frequency of the *bla*_NDM_,  *bla*_PER_,  *bla*_VEB_,  *bla*_IMP_, and *bla*_VIM_ type genes among* A. baumannii* strains isolated from patients admitted to Loghman Hakim and Milad hospitals, Tehran, Iran, from year 2012 to 2013.

## 3. Patients and Methods

### 3.1. Bacterial Identification

From June 2012 to May 2013, one hundred and eight nonduplicate nonconsecutive isolates of* A. baumannii* were recovered from blood, wound, urine, sputum, and respiratory tract of patients from 2 hospitals (fifty-eight isolates belonged to Loghman Hakim and fifty isolates to Milad hospitals) in Tehran, Iran. The isolates were identified by conventional biochemical methods [2] and also confirmed for* blaOXA-51* gene by PCR.

### 3.2. Antimicrobial Susceptibility Testing

Antimicrobial susceptibility test to imipenem (IPM: 10 *μ*g), meropenem (MEM: 10 *μ*g), ceftazidime (CAZ: 30 *μ*g), cefotaxime (CTX: 30 *μ*g), amikacin (AK: 30 *μ*g), piperacillin/tazobactam (PTZ: 100/10 *μ*g), piperacillin (PIP, 100 *μ*g), ampicillin (AMP, 10 *μ*g), tetracycline (TE, 10 *μ*g), colistin sulphate (CT, 10 *μ*g), ciprofloxacin (CIP: 5 *μ*g), cefepime (FEP: 30 *μ*g), trimethoprim-sulfamethoxazole (TS, 2.5 *μ*g), and gentamicin (GEN: 10 *μ*g) (Mast, UK) was performed by the Kirby-Bauer disk diffusion method on Mueller Hinton agar (Merck, Germany) based on Clinical Laboratory Standards Institute (CLSI) Guidelines 2012 [11].* Escherichia coli* ATCC 25922 was used as the quality control strain.

### 3.3. Minimum Inhibitory Concentration (MIC)

Strains resistant to imipenem, meropenem, ceftazidime, cefepime, cefotaxime, and colistin using the disk diffusion test were rechecked by the broth microdilution method according to the guidelines of the CLSI 2012 [11].

### 3.4. Phenotypic Detection of MBL

Combined disk diffusion test (CDDT) was performed for identification of MBLs by imipenem and meropenem (Mast Group, Merseyside, UK) alone and in combination with EDTA. An increase in zone diameter of ≥7 mm around the Imipenem+EDTA and Meropenem+EDTA disks compared to that of Imipenem and Meropenem disks alone, respectively, was considered positive for MBL production [12].

### 3.5. Phenotypic Detection of ESBL

Detection of ESBLs was tested for all the isolates by combination disk diffusion test (CDDT) containing ceftazidime (CAZ) and cefotaxime (CTX) alone and with CAZ 30 *μ*g + clavulanic CA 10 *μ*g and CTX 30 *μ*g + clavulanic CA 10 *μ*g per disc (Mast Group, Merseyside, UK). The zones of inhibition were compared with the CTX and CAZ discs alone and compared with the CAZ 30 *μ*g + clavulanic (CA) 10 *μ*g and CTX 30 *μ*g + CA 10 discs. An increase in zone diameter of ≥5 mm in the presence of clavulanic acid indicated the presence of ESBL in the test organism.* Escherichia coli* ATCC 25922 and* Klebsiella pneumoniae* ATCC700603 were used as negative and positive controls for ESBL production, respectively [11].

### 3.6. DNA Extraction

Total DNAs of the different bacterial isolates were extracted by the DNA extraction kit (Bioneer Company, Korea, Cat. number K-3032-2).

### 3.7. Detection of *bla*_NDM_,* bla*_PER_,* bla*_VEB_,* bla*_IMP_, and* bla*_VIM_ Genes by PCR

PCR was used for screening of the *bla*_NDM_, *bla*_PER_,   *bla*_IMP_,   *bla*_VIM_, and *bla*_VEB_ genes. The primers used for *bla*_NDM_,   *bla*_PER_, *bla*_VEB_, *bla*_IMP_, and *bla*_VIM_ were as follows: NDM-F (5′-GGTTTGGCGATCTGGTTTTC-3′) and NDM-R (5′-CGGAATGGCTCATCACGATC-3′) for *bla*_NDM_; PER-F (5′-GCAACTGCTGCAATACTCGG-3′) and PER-R (5′-ATGTGCGACCACAGTACCAG-3′) for *bla*_PER_; VEB-F (5′-CGACTTCCATTTCCCGATGC-3′) and VEB-R (5′-GGACTCTGCAACAAATACGC-3′) for *bla*_VEB_; IMP-F (5′-GAAGGCGTTTATGTTCATAC-3′) and IMP-R (5′-GTAAGTTTCAAGAGTGATGC-3′) for *bla*_IMP_; VIM-F (5′-GATGGTGTTTGGTCGCATA-3′) and VIM-R (5′-CGAATGCGCAGCACCAG-3′) for *bla*_VIM_. The PCR mixture contained the DNA template, forward/reverse primers, and master mix (Bioneer Company, Korea, Cat. number K-2016). Amplification was carried out with the following thermal cycling conditions: 5 minutes at 94°C and 36 cycles of amplification consisting of 1 minute at 94°C, 1 minute at 52–56°C, and 1 minute at 72°C, with 5 minutes at 72°C for the final extension. PCR product bands were analyzed after electrophoresis on a 1% agarose gel at 95 V for 45 minutes in 1X TBE containing ethidium bromide and the result was checked under UV irradiation.

### 3.8. Sequencing Method

The PCR purification kit (Bioneer Co., Korea) was used to purify PCR products and sequencing was performed by the Bioneer Company (Korea). The nucleotide sequences were analyzed with the Chromas 1.45 software and the BLAST program from the National Center for Biotechnology Information website (http://www.ncbi.nlm.nih.gov/BLAST).

### 3.9. Statistical Analysis

This research was a descriptive-application study. MINITAB16 software was used for statistical analyses. The *P* value and confidence of intervals were <0.05 and 95%, respectively.

## 4. Results

**Figure 1:**
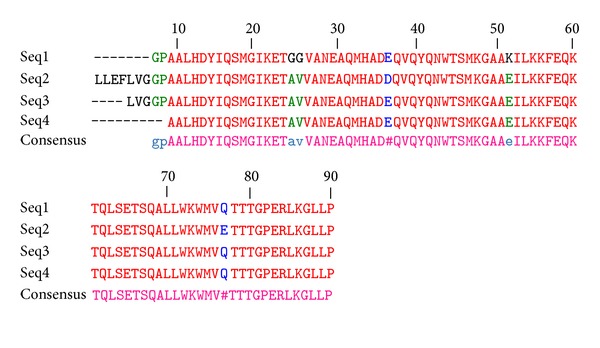
Multiple sequence alignment (seq1 related to Milad hospital and seq2, seq3, and seq4 related to Loghman Hakim hospital).

Fifty-eight strains were isolated from Loghman Hakim Hospital (53.7%) and fifty from Milad Hospital (46.29%). Fifty-one strains were isolated from female patients (47.2%) and fifty-seven from males (52.8%). Of the 108 isolates, 29 (26.9%) were isolated from urine, 4 (3.7%) from wound, 57 (52.8%) from tracheal tube, 8 (7.4%) from blood, 8 (7.4%) from pleural fluid, and 2 (1.9%) from other samples. The age range of the patients was 1 to 90 years. The isolates were obtained from patients in different age groups: 2–29 years (*n* = 10), 30–39 (*n* = 14), 40–49 years (*n* = 21), 50–59 years (*n* = 16), 60–69 (*n* = 24), and 70–79 years (*n* = 17), and six isolates were isolated from patients of more than eighty years of age. The resistance of* A. baumannii* isolates to tested antibiotics was 108 (100%) to cefotaxime, 103 (95.4%) to ceftazidime, 99 (91.7%) to meropenem, 99 (91.7%) to imipenem, 44 (40.7%) to gentamicin, 87 (80.6%) to amikacin, 100 (92.6%) to ciprofloxacin, 105 (95.7%) to cefepime, 105 (97.2%) to piperacillin, 103 (95.4%) to piperacillin/tazobactam, 106 (98.1%) to ampicillin/sulbactam, 106 (98.1%) to cotrimoxazole, 87 (80.6%) to tetracycline, and 1 (1.8%) to colistin (Table 1). The results of MIC test of* different antibiotics on A. baumannii* isolates are shown in Table 2. By using the combined disk diffusion test (CDDT), it was found that among 99 imipenem nonsusceptible* A. baumannii* strains, 86 (86.86%) were MBL producers and out of 108 cefotaxime-nonsusceptible* A. baumannii* strains, 91 (84.2%) were ESBL producers. The prevalence of *bla*_PER-1_ and *bla*_VEB-1_ genes among 91 ESBL-producing* A. baumannii* isolates was 71 (78.03%) and 36 (39.5%), respectively, and for IMP-1 and VIM-1 genes among metallo-beta-lactamase-producing* A. baumannii* isolates it was 3 of 86 (3.48%) and 15 of 86 (17.44%), respectively. *bla*_OXA-51_ has been investigated and was detected in all isolates. Fortunately, *bla*_NDM_ gene was not detected in isolates. Sequencing of PCR products showed conserved regions for the restriction sequence *bla*_PER-1_, *bla*_IMP_, *bla*_VIM_, and *bla*_VEB-1_ genes which was confirmed by BLAST in NCBI. The nucleotide sequence data reported in this paper have been submitted to the GenBank sequence database and assigned accession numbers KF723587, KF723588, KF723589, and KF723590 for *bla*_PER-1_ gene, KF723586 for *bla*_VEB-1_ gene, and KF723585 for *bla*_IMP-1_. The sequences of *bla*_PER-1_ gene in* A. baumannii* strains isolated from Loghman Hakim hospital were 100% the same but were not similar to the sequences of *bla*_PER-1_ gene in* A. baumannii* strains isolated from Milad hospital (http://multalin.toulouse.inra.fr/multalin/multalin.html) (Figure 1).

## 5. Discussion

*A. baumannii* is responsible for hospital-acquired infections and has recently become one of the most important healthcare-associated infections in hospitals. Infection caused by this bacterium often leads to significant mortality and morbidity [13]. Many researchers have reported the outbreak of PDR* A. baumannii*. Although* A. baumannii* is an increasingly common nosocomial pathogen that can cause serious infections in intensive care units [14], resistance rates of isolates were as follows: 103 (95.4%) to ceftazidime, 99 (91.7%) to meropenem, 99 (91.7%) to imipenem, 44 (40.7%) to gentamicin, 87 (80.6%) to amikacin, 100 (92.6%) to ciprofloxacin, 105 (95.7%) to cefepime, 105 (97.2%) to piperacillin, 103 (95.4%) to piperacillin/tazobactam, 106 (98.1%) to ampicillin/sulbactam, 106 (98.1%) to cotrimoxazole, 87 (80.6%) to tetracycline, and 1 (1.8%) to colistin. Also, no susceptible isolate to cefotaxime was detected. So, the best coverage against the study isolates was obtained with colistin sulphate and gentamicin. Based on different studies, it is clear that emergence of resistant* A. baumannii* strains is increasing worldwide [2]. These studies showed that the PDR strains were resistant not only to beta lactams, including the third generation of cephalosporins and carbapenems, but also to other drug categories including aminoglycosides and fluoroquinolones. Another concerned problem is related to their multidrug resistance which restricts the treatment procedure [2]. Resistance to beta-lactams is related to various enzymes that are produced, including extended spectrum beta-lactamases (ESBLs) and metallo-beta-lactamases (MBLs) which belong to Ambler A and B divisions [6, 15]. In this study by using the CDDT method, 86 (86.86%)* A. baumannii* isolates were identified as MBL producers. Safari et al. reported that the resistance rates of* A. baumannii* isolates were 85%, 94%, 97%, 84%, 95%, and 98% against imipenem, meropenem, ciprofloxacin, amikacin, piperacillin/tazobactam, and cefotaxime, respectively. Results of *E*-test MBL illustrated that 99% of all isolates were MBL producers [16]. Peymani et al. reported that among 63 carbapenem nonsusceptible* A. baumannii *isolates, 31 (49%) were found to be MBL producers [17]. Most of the time, the MBL producers can hydrolyze a wide range of antibiotics except aztreonam [18]. Usually, restrictions in phenotypic methods make researchers confirm phenotypic results by using molecular methods. On the other hand, there are different genes which encode the beta-lactamases. Among MBL genes, IMP is more important especially in Iran [19]; however, its first report was from Japan in 1980 [12]. The other gene is VIM which was reported before from Ahwaz, another city of Iran [12]. In our study, the IMP enzyme was identified only in three* A. baumannii* strains and VIM gene was detected among seventeen* A. baumannii* strains by using PCR and further sequencing which may be related to differences in the time of studies and consequently changes in antibiotics prescription or used primers [20]. The MBL coding gene is *bla*_NDM-1_ which was identified recently and reported from New Delhi, India, for the first time and after that from other countries including Pakistan. The close distance of these countries to Iran and large number of trips between the countries on one side and the ease of resistance transfer among bacteria on the other hand led us to think that it may be probable for our isolates to have the same gene. These kinds of studies are valuable to prevent distribution of resistant bacteria to other parts of the world. Finally, by accurate MBL enzyme screening and further precise supervision of the hospital practitioners it will be possible to control the spread of multidrug resistant* A. baumannii* strains and decrease related infections especially in ICU patients. Based on the available data,* A. baumannii* are growing to become the most important common ESBL producing bacteria and consequently making their eradication difficult. In this study, 91 (84.2%) of* A. baumannii* were identified as ESBL producers by phenotypic tests, which was more than Owlia et al.'s study (21%), Farajnia et al.'s study in Iran (70%), and another study in Poland (20%) [15, 21]. The high rate of ESBL prevalence in Iran and its widespread dissemination is cause of worry. In this study, the prevalence of *bla*_PER-1_ genes among 91 of ESBL-producing* A. baumannii* isolates was 71 (78.03%). According to the present study PER-1 is the most common ESBL genotype among* A. baumannii* strains which is inconsistent to other studies that showed different prevalence of PER-1. The prevalence of this genotype was reported 51% in Iran, 46% in Turkey and 54.6% in South Korea. Screening for VEB genotype revealed that 36 (39.5%) of* A. baumannii* isolates contained VEB-1 gene. The prevalence of this gene was reported to be 10% in Iran and 47.61% in the USA [10]. The prevalence of *β*-lactamase-producing isolates and their isolation from life-threatening infections is dramatically increasing worldwide. Intensity pressure for usage of antimicrobial drugs by patients resulted in eradication of normal flora and situation of MDR isolates substitution. This study showed that *β*-lactamase producing* A. baumannii* strains are an emerging threat in ICUs and should be supervised by implementation of timely identification and strict isolation methods that will help to reduce their severe outcomes and mortality rate of patients.

## Figures and Tables

**Table 1:** Antimicrobial susceptibility test results of 108 isolated *Acinetobacter baumannii*.

Antibiotic	Resistant, number (%)	Intermediate, number (%)	Sensitive, number (%)
Gentamicin	44 (40.7)	7 (6.5)	57 (52.8)
Ampicillin/sulbactam	106 (98.1)	0 (0.0)	2 (1.8)
Amikacin	87 (80.6)	4 (3.7)	17 (15.7)
Imipenem	99 (91.7)	3 (2.8)	6 (5.6)
Cefotaxime	108 (100)	0 (0.0)	0 (0.0)
Cefepime	105 (95.7)	2 (1.8)	1 (1.8)
Piperacillin	105 (97.2)	2 (1.8)	1 (0.9)
Ciprofloxacin	100 (92.6)	1 (0.9)	7 (6.5)
Meropenem	99 (91.7)	0 (0.0)	9 (8.3)
Piperacillin/tazobactam	103 (95.4)	1 (1.8)	4 (3.7)
Ceftazidime	103 (95.4)	0 (0.0)	5 (4.7)
Co-trimoxazole	106 (98.1)	0 (0.0)	2 (1.8)
Tetracycline	87 (80.6)	9 (8.3)	12 (11.1)
Colistin	2 (1.8)	0 (0.0)	106 (98.2)

**Table 2:** Minimum inhibitory concentration of different antimicrobial agents among 108 *Acinetobacter baumannii* isolates.

Antibiotics	MIC (*μ*g/mL)		
	Range	MIC_50_	MIC_90_
Meropenem	1–256	32	128
Imipenem	2–256	128	256
Ceftazidime	2–>512	256	512
Cefepime	1–256	64	128
Cefotaxime	2–>512	256	512
Colistin	0.25–128	≤1	2
